# FRTL-5 Rat Thyroid Cells Release Thyroglobulin Sequestered in Exosomes: A Possible Novel Mechanism for Thyroglobulin Processing in the Thyroid

**DOI:** 10.1155/2016/9276402

**Published:** 2016-06-09

**Authors:** Pavel Vlasov, Sonia Q. Doi, Donald F. Sellitti

**Affiliations:** Department of Medicine, Divisions of Endocrinology and Nephrology, Uniformed Services University of the Health Sciences, Bethesda, MD 20814-4799, USA

## Abstract

Exosomes are 30–100 nm, membrane-bound vesicles containing specific cellular proteins, mRNAs, and microRNAs that take part in intercellular communication between cells. A possible role for exosomes in thyroid function has not been fully explored. In the present study, FRTL-5 rat thyroid cells were grown to confluence and received medium containing either thyroid stimulating hormone (TSH), exogenous bovine thyroglobulin (bTg), or neither additive for 24 or 48 hours followed by collection of spent medium and ultracentrifugation to isolate small vesicles. Transmission electron microscopy and Western blotting for CD9 indicated the presence of exosomes. Western blotting of exosome extract using a monoclonal anti-Tg antibody revealed a Tg-positive band at ~330 kDa (the expected size of monomeric Tg) with a higher density in TSH-treated cells compared to that in untreated cells. These results are the first to show that normal thyroid cells in culture produce exosomes containing undegraded Tg.

## 1. Introduction

Thyroglobulin (Tg) transport through the thyroid follicular cell into the follicular lumen (and to a more limited degree across the basement membrane and into the circulation) involves a number of plasma membrane receptors and internal transport systems that direct the Tg molecule to specific intracellular and extracellular locations [1, 2]. Although internal Tg transport takes place within membrane-bounded organelles (e.g., endosomes and Golgi), Tg transport outside the cell has heretofore been thought to encompass only the secretion of soluble Tg through extracellular domains such as the thyroid follicular lumen, extracellular spaces, and the circulatory system [1].

Several independent findings [3–5] taken together however suggest that Tg might also be secreted from thyroid cells as a constituent of membrane-delimited vesicles (exosomes) that originate as invaginations of late stage endosomes called multivesicular bodies (MVBs) [6]. Exosomes together with microvesicles that bud directly from the plasma membrane into the extracellular space contain proteins, mRNAs, and microRNAs that are increasingly being seen as performing important regulatory roles in both normal and abnormal (e.g., cancerous) cells [7]. No specific role for either type of vesicle in the thyroid gland has yet been defined.

The production of exosomes in thyroid-derived cells was indicated in a recent study [3] showing that three thyroid cancer cell lines (all derived initially from thyroid follicular cells) release vesicles with the well-defined morphological features of exosomes into the cellular environment. However, many tumor cell types have been shown to secrete large numbers of exosomes differing in both protein and nucleic acid content from exosomes released from the cell type of origin [6, 8, 9], so it would be difficult to draw conclusions from the cancer cells as to what exosomes contain and what they do in the normal thyroid. However, a recent proteomic analysis of fetal bovine serum-derived exosomes listed Tg as one of the 51 different proteins contained in an exosome-enriched fraction of the serum, but not in an exosome-free fraction [4]. This finding would support the hypothesis that thyroglobulin-containing exosomes may be released from normal thyroid cells into the circulation in healthy individuals. On the other hand, a TEM study of the thyroid of Risso's dolphin,* Grampus griseus* [5], showing circular membrane-bound vesicles <100 nM in size located within the lumen of thyroid follicles sufficiently distant from the epithelium so as not to represent microvilli might be evidence of vesicle secretion into the colloid follicle, similar to what has been shown to occur in the ovarian follicle [10].

In the present study we show for the first time that a line of functional thyroid cells (FRTL-5) secretes Tg-containing exosomes into the culture medium, suggesting that the release of Tg in exosomes could be a normal physiologic process that serves as an alternative to currently understood pathways of Tg secretion and processing.

## 2. Materials and Methods

### 2.1. FRTL-5 Cell Culture

A continuous, diploid cell line, FRTL-5, derived from the thyroid gland of the Fisher rat and retaining many of the biochemical markers of the thyroid follicular cell (such as TSH dependence) was employed in this study [11]. The cells were grown in a medium (6H medium) consisting of Coon's Modified Ham's F-12 solution containing six critical hormones (insulin (10 *μ*g/mL), transferrin (5 *μ*g/mL), somatostatin (0.01 *μ*g/mL), glycyl-L-histidyl-L-lysine acetate (0.1 *μ*g/mL), hydrocortisone (0.362 ng/mL), and thyroid stimulating hormone (TSH) (0.001 IU/mL)) plus 5% fetal bovine serum, glutamine, penicillin, and streptomycin. Medium containing all of these components is referred to as 6H medium, and medium with all components except TSH is referred to as 5H medium [12].

To collect exosomes for TEM, FRTL-5 cells were grown in plastic 75 cm^2^ culture flasks containing 10 mL of 6H medium at 37°C in the presence of 5% CO_2_ in a humidified incubator. Medium was replaced every 3 days until cells reached confluence, at which point the cultures were trypsinized and split into four flasks containing 6H media. Samples of spent 6H media were collected and stored at −80°C for later exosome collection and TEM.

For Western blot, confluent FRTL-5 cultures in 6, 75 cm^2^ flasks were washed with HBSS and received 5H medium for 3 days, after which 3 cultures received a volume of 7 mL of 5H medium and the remaining 3 received 7 mL of 6H medium. Medium was collected after 24 h and cells were trypsinized and pelleted. Media samples and cell pellets were stored at −80°C.

### 2.2. Exosome Isolation

For all studies, purified exosomes were collected from medium using two steps of ultracentrifugation as described previously [13]. Briefly, conditioned medium was removed from FRTL-5 cultures, a protease inhibitor cocktail (complete protease inhibitor cocktail tablets, Roche Diagnostics) was added, and the medium was centrifuged at 1500 rpm for 10 min to remove cellular debris. The supernatant was then frozen at −80°C until exosome isolation. The cellular monolayer was then washed and trypsinized, and cell counts were determined with a hemocytometer.

To isolate microvesicles, the frozen medium was thawed and centrifuged at 17,000 ×g for 18 min to pellet larger organelles and other membrane structures out, followed by a final centrifugation at 200,000 ×g for 1 hour and 15 minutes and collection of the pellet (exosomal fraction) for either electron microscopy or protein extraction. For some studies, additional pellets and supernatants from the two centrifugation steps were collected for protein measurement and Western blot. For some Western blot studies, exosome collection from 5H and 6H medium, respectively, was maximized by the sequential ultracentrifugation of medium collected from each of three flasks in the same centrifuge tube, adding medium to the pellet from the previous round of ultracentrifugation. Exosome pellets for Western blot were resuspended in 15 *μ*L of lysis buffer containing leupeptin and phenylmethylsulfonyl fluoride (PMSF) and frozen at −4°C. Exosome pellets intended for TEM were resuspended in phosphate-buffered saline.

### 2.3. Transmission Electron Microscopy (TEM)

Approximately 5 *μ*L of exosome sample was applied to the surface of Electron Microscopy Sciences formvar-coated, carbon-stabilized 3 mm copper grids. Each sample was left on a grid for 5 minutes before the excess liquid was wicked off using the edge of a piece of filter paper. Next, 5 *μ*L of Electron Microscopy Sciences 2% aqueous uranyl acetate stain was applied to the grid for 1 minute as a negative stain, excess solution was wicked off, and the samples were allowed to air-dry before observation. Imaging was done using a JEOL JEM-1011 transmission electron microscope and images were collected using an Advanced Microscopy Techniques XR50S-A digital camera.

### 2.4. SDS-PAGE and Western Blot

Exosome samples from 5H and 6H cells were mixed with 15 *μ*L of Bio-Rad 2x Laemmli Buffer. 9.25 *μ*L supernatant samples were mixed with 3.75 *μ*L of NuPAGE LDS Sample Buffer (4x concentration) containing lithium dodecyl sulfate and 1.5 *μ*L of NuPAGE Sample Reducing Agent (10x concentration) containing 500 mM dithiothreitol (DTT). All samples were heated to 70°C for 10 minutes and 15 *μ*L of each sample was loaded into lanes in a precast polyacrylamide 4–12% gel alongside a Bio-Rad Precision Plus Protein Kaleidoscope Standard (Bio-Rad Laboratories). Samples on the gel were then transferred to a PDVF membrane using a dry transfer system (iBlot, Thermo Fisher Scientific). The membrane was blocked with 5% fat-free milk/0.05% Tween-20 in immuno-TRIS. The exosome marker CD9 was detected using a rabbit polyclonal antibody (System Biosciences) and a goat anti-rabbit IgG antibody conjugated to horseradish peroxidase (HRP). Antibodies were each applied to the membrane dissolved in 10 mL of blocking solution with the primary antibody applied overnight for approximately 24 hours and the secondary antibody applied for 1 hour. SDS-PAGE procedures used were developed by Dr. Sonia Q. Doi as an optimization of existing SDS-PAGE and Western blotting procedures. Positive signal was detected with addition of a substrate (SuperSignal West Femto chemiluminescence reagent, Fisher Scientific) and the image was captured by LCD camera system. Subsequently, the membrane was stripped of antibodies using a buffer containing 3-(N-morpholino)propanesulfonic acid (MOPS), *β*-mercaptoethanol, and SDS. A second immunostaining procedure was performed to detect thyroglobulin (Tg) using a Dako mouse anti-human-Tg clone DAK-Tg6 as a primary antibody (Dako, Agilent Technologies) and a horse anti-mouse IgG conjugated with HRP as the secondary antibody. The blots were developed with a SuperSignal West Pico chemiluminescence kit and imaged as described above.

## 3. Results

### 3.1. Transmission Electron Microscopy

**Figure 1:**
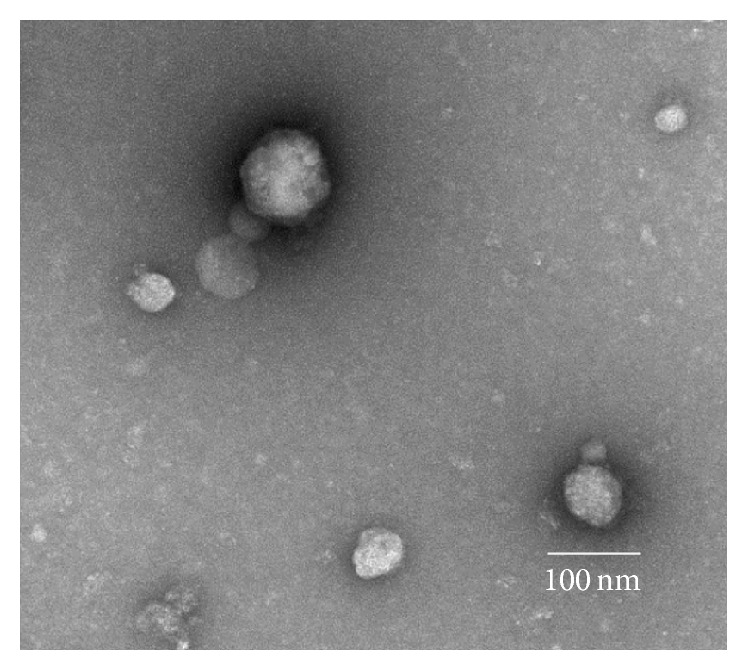
Transmission electron micrograph of a sample of the pellet obtained by ultracentrifugation of Coon's Modified Ham's F-12, 6-hormone medium used to grow FRTL-5 cells. Objects in the frame exhibit the characteristic shape of microvesicles under transmission electron microscopy, a spherical shape between 30 and 100 nm in diameter.

Exosomes collected by ultracentrifugation of conditioned FRTL-5 6H growth medium were examined using TEM of resuspended pellet contents immobilized and stained on copper grids (Figure 1). Images show spherical structures of varying sizes primarily less than 100 nm in diameter, consistent with the size and shape of exosomes.

### 3.2. Western Blot for Thyroglobulin

**Figure 2:**
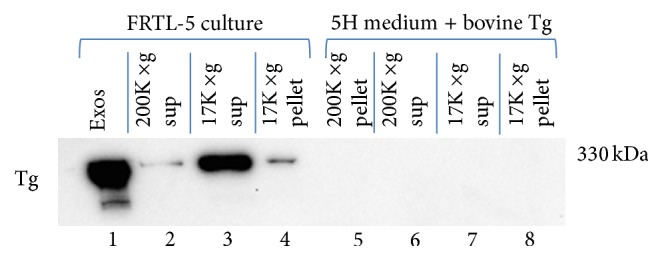
SDS-PAGE and Western blot with anti-Tg of samples from different stages of exosome isolation by ultracentrifugation: 17,000 ×g pellet and supernatant, 200,000 ×g supernatant, and 200,000 ×g (exosome pellet) from 6H medium collected from a confluent culture of FRTL-5 cells (lanes 1–4) and fresh 5H medium with 1 mg/mL bovine Tg added (lanes 5–8). Western blots were treated with a monoclonal anti-human-Tg antibody and developed using SuperSignal West Pico. “Exos” = exosomes (i.e., 200,000 ×g pellet).

Figure 2 shows the results of a representative Western blot for Tg on pellet and supernatant fractions collected by the differential centrifugation of 6H medium exposed to FRTL-5 cells in culture for 48 h (lanes 1–4) and from fresh 5H medium containing 1 mg/mL exogenous* bovine* Tg (bTg) (lanes 5–8). Pellets and supernatants were saved following both 17,000 ×g (low-speed) and 200,000 ×g (high-speed) centrifugations and proteins from both low-speed and high-speed (i.e., exosome) pellets were extracted and subjected to electrophoresis together with samples of the low-speed and high-speed (i.e., soluble Tg) supernatants. The gels were then transblotted and Western blot immunostaining for Tg was performed.

The figure clearly shows the presence of immunoreactive bands at the expected size of ~330 kDa for monomeric Tg in each of the samples taken from medium exposed to FRTL-5 cells (lanes 1–4). The intensity of the bands varied considerably, due to the intrinsic differences in protein concentration between supernatants and pellet extracts and also, as we demonstrate below, because of differential partitioning of Tg into either a soluble state (supernatant) or membrane-delimited exosomes (pellet) after the final centrifugation. The 200,000 ×g exosome pellet also exhibited a less dense band of 220 kDa (Figure 2).

In contrast to the conditioned medium collected from FRTL-5 cells (lanes 1–4), fresh 5H medium to which 1 mg/mL bTg was added did not display any 330 kDa immunoreactive bands (lanes 5–8), confirming that the Dako anti-human-Tg monoclonal antibody used in this study recognizes rat but not bovine Tg (Figure 2). The species specificity of the antibody was advantageous for subsequent experiments to examine the effects of exogenous Tg and TSH on the secretion of exosomal Tg from FRTL-5 cells.

**Figure 3:**
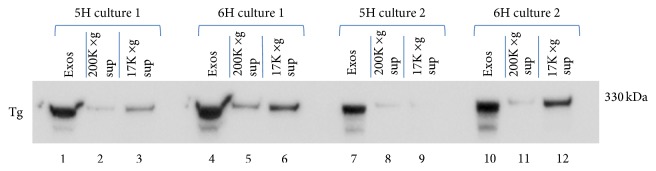
SDS-PAGE and Tg Western blot of samples from different stages of exosome isolation by ultracentrifugation: 17,000 ×g supernatant, 200,000 ×g supernatant, and 200,000 ×g (exosome pellet) from samples of 5H (lanes 1–3 and 7–9) and 6H media (lanes 4–6 and 10–12) collected from confluent cultures of FRTL-5 cells incubated for 48 hours. 15 *μ*L samples of resuspended isolated exosomes were loaded for 5H culture 1 and 6H culture 1, while 7 *μ*L samples were loaded for both 5H culture 2 and 6H culture 2. Due to the limited number of lanes in each gel, lanes numbered 1 through 6 and 7 through 12 were run on separate gels. Protein concentrations of resuspended exosome samples were nearly identical in both of the two 5H cultures and both of the 6H cultures. Western blots were treated with a monoclonal anti-human-Tg antibody and developed using SuperSignal West Pico. “Exos” = exosomes (i.e., 200,000 ×g pellet).

In order to gain a better understanding of Tg localization into exosomes under high- and low-TSH conditions, the partitioning of Tg into pellet and supernatant fractions from 5H- and 6H-treated cells as illustrated in Figure 2 was also examined in two additional studies (Figure 3), in which careful measurements of total protein were made.

As noted above, the 200,000 ×g pellets showed strong Tg immunoreactivity at 330 kDa and also to a lesser degree at 220 kDa, with the 6H extracts yielding bands of greater density than in the corresponding 5H extracts (Figure 3). Immunopositive Tg bands were also observed in both 17,000 ×g and 200,000 ×g supernatants, but these were of lower density than in the final pellet due to the lower concentration of protein loaded in supernatant fractions than in pellet fractions.

**Figure 4:**
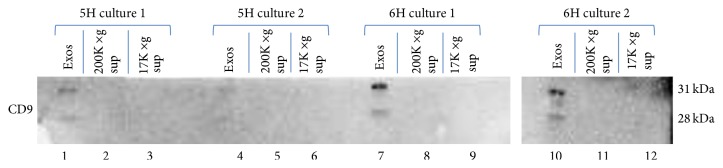
SDS-PAGE and Western blotting of samples from different stages of exosome isolation by ultracentrifugation: 17,000 ×g supernatant, 200,000 ×g supernatant, and 200,000 ×g (exosome pellet) from samples of 5H (lanes 1–6) and 6H media (lanes 7–12) collected from confluent cultures of FRTL-5 cells incubated for 48 hours. Western blots were treated with a monoclonal anti-CD9 antibody and developed using SuperSignal West Femto. All exosome samples contained 15 *μ*L of resuspended pellet from the 200,000 ×g ultracentrifugation. 5H samples exhibited small, barely detectable bands for CD9 though some CD9 was present, while 6H samples exhibited much stronger CD9 signals indicating larger amounts of exosomes. “Exos” = exosomes (i.e., 200,000 ×g pellet).

To ascertain the presence or absence of exosomes in each of these extracts, Western blots for the exosome marker CD9 were also performed (Figure 4). Immunoreactive bands of appropriate molecular weight for CD9 (two bands of 31,000 and 28,000 D, resp.) were observed only in the 200,000 ×g pellet and were not observed in either the 200,000 ×g supernatant or the 17,000 ×g pellet. Moreover, the results indicated that the amount of CD9 was greater in the 6H samples than in the 5H samples (Figure 4).

Since the protein concentration of exosomal pellets differed between 5H- and 6H-treated cells, we normalized Tg exosomal content (measured in uncalibrated optical density) to total protein content in order to make valid comparisons of total Tg present in the final 200,000 ×g pellet of cells grown in the presence or absence of TSH (Table 1). Results showed an approximately 2-fold greater Tg content in exosomes from the 6H-treated cells than from 5H-treated cells. This finding suggests that higher Tg content in the 200,000 ×g pellet from 6H cells could represent not only a greater number of exosomes secreted from 6H exposed cells, but also selective partitioning of Tg into these exosomes compared to exosomes collected from 5H exposed cells.

Recent studies have shown that Tg exerts an autoregulatory effect on its own production in FRTL-5 cells. We were therefore interested in ascertaining whether treatment of cells with a high content of bovine Tg (bTg) could markedly affect the amount of rat Tg (rTg) partitioned into exosomes.

**Figure 5:**
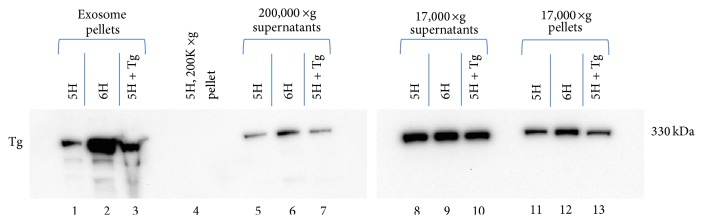
SDS-PAGE and Tg Western blot of samples from different stages of exosome isolation by ultracentrifugation: 17,000 ×g pellet and supernatant, 200,000 ×g supernatant, and 200,000 ×g (exosome pellet) from samples of 5H medium (lanes 1, 5, 8, and 11), 6H medium (lanes 2, 6, 9, and 12), and 5H medium with 1 mg/mL Tg (lanes 3, 7, 10, and 13) from confluent cultures of FRTL-5 cells incubated for 48 hours. Additionally, lane 4 contains a sample of the 200,000 ×g pellet obtained from fresh 5H media (i.e., unexposed to FRTL-5 cells). Western blots were treated with a monoclonal anti-human-Tg antibody and developed using SuperSignal West Pico. “Exosome pellets” = 200,000 ×g pellets.

Figure 5 shows the results of such a study in which FRTL-5 cells were exposed to either 6H medium, 5H medium containing 1 mg/mL bTg, or 5H medium only (control) for 48 h prior to collection of the medium for exosome preparation. Equal volumes of supernatant from the 17,000 ×g centrifugation, theoretically containing both soluble protein and microvesicle/exosomal fractions, produced definitive immunoreactive Tg bands of ~330 kDa that showed little difference in density among the three treatment groups (Figure 5, lanes 8–10). Equal volumes of extract from the low-speed pellet, theoretically containing particles larger than exosomes and other high MW particulate matter, showed somewhat more variability in Tg content than the corresponding pellets, with the TSH-treated group showing a 2-fold higher density band than the 5H group (Figure 5, lanes 11–13).

In contrast to the low-speed pellets, equal volumes of extract from the 200,000 ×g pellet from 5H-treated, 6H-treated, and bovine Tg-treated cells, respectively (Figure 5, lanes 1–3), showed marked variability, with the TSH-treated (6H) exosome extract exhibiting an approximately 10-fold greater density than the 5H extract. The relevant band from the Tg-treated exosome extract was intermediate in density between the other two groups. The bovine Tg-treated sample also showed “funneling” and band distortion of the type associated with an overload of total protein in the sample (lane 3). In contrast to the exosome fractions, Tg in the 200,000 ×g supernatants showed less variation, but the TSH-treated group exhibited a denser Tg band than the other two groups (Figure 5, lanes 5–7).

## 4. Discussion

The present study demonstrates, using transmission electron micrography and Western blotting for CD9, that exosomes are produced* in vitro* by cultures of thyroid-derived cells. In addition, Western blotting using a monoclonal antibody against Tg suggests that Tg is encapsulated in these vesicles and released into the culture medium together with Tg secreted via conventional exocytosis.

TEM images of microvesicle pellets from conditioned 6H media consistently showed spherical structures within the expected exosomal size range of 30 to 100 nm in diameter. It is likely that some of these structures are exosomes, but the presence of other possible structures in these samples with similar sizes and shapes (including spherical protein aggregates and other membrane-delimited vesicle structures) cannot be excluded using this technique. Western blotting for exosome markers supports the presence of exosomes in these samples, but the definitive identities of the observed spherical structures will require further TEM imaging with immunogold staining for exosome markers.

The results suggest that sequestration of Tg into exosomes could represent a normal alternative pathway of Tg processing in the thyroid cell, in which Tg is diverted from the pathway of lysosomal degradation and production of T_3_ and T_4_, and released intact from the cell [1]. Given the novelty of this finding, alternative trivial explanations for these results must also be considered, including the coprecipitation of insoluble Tg with exosomes and the nonspecific attachment of Tg to those exosomes.

Colloidal Tg is known to be covalently linked by different enzymatic reactions in different species to form insoluble high molecular weight aggregates (globules) of Tg that could conceivably coprecipitate with exosomes [14]. Indeed the Tg obtained from bovine thyroid glands used in this study contained a substantial fraction (11%, according to the manufacturer) of cross-linked, insoluble Tg [15] that probably accounted for the large yellowish pellets collected after the final 200,000 ×g centrifugation of either conditioned medium from bTg-treated cells or fresh medium to which 1 mg/mL exogenous bTg was added. Since cross-linkage of Tg into insoluble forms occurs only within the thyroid follicle [12], monolayer FRTL-5 medium would have contained very little, if any, insoluble* rat* Tg (rTg) to coprecipitate with exosomes. Moreover, had rat Tg been adsorbed to other proteins in high concentration in the incubation medium, most especially the 1 mg/mL bTg that was added in some studies, then the exosome fraction from cells treated with bTg (Figure 5, exosome pellet, lane 3) should have contained a much greater amount of 330 KDa immunoreactive rTg than the other two treatments, acquired through nonspecific adsorption to and coprecipitation of soluble rTg with bTg.

Previous investigations from this lab and others have shown a suppressive effect of excess exogenous bTg on Tg production by FRTL-5 cells that we have argued constitutes a local negative feedback system [16]. However, no clear effect of that exogenous Tg on “exosomal” rTg could be discerned from a comparison of the density of Tg staining in the exosomes of control, TSH-treated, and Tg-treated cells shown in Figure 5. An autocrine regulatory effect of Tg on the packaging or secretion of Tg in exosomes thus cannot be inferred. For the reasons discussed above, however, the experiment provides support for the hypothesis that most of the Tg in the exosome pellet is contained within vesicles and not attached to aggregate surfaces.

Unlike exogenous Tg, an effect of TSH on exosomal secretion from thyroid cells has substantial support from the present Western blot results which show consistently that exosome extracts from cells receiving TSH had a higher content of immunoreactive Tg than cells lacking Tg. Moreover, our results suggest that the effect of TSH may be due to a greater content of Tg per exosome (twofold) in addition to the secretion of a higher number of exosomes over a given time period.

Our current study of exosomes in FRTL-5 cells in monolayer culture does not distinguish between apical and basal secretion of the vesicles. Investigations of exosomes in polarized cultures of FRTL-5 cells will be important in addressing this question definitively. The previous identification of membrane-bound vesicles of a size consistent with exosomes in the follicle lumen in TEM studies [11] suggests that at least some exosomes are secreted into the follicular colloid. Exosome secretion across the basement membrane and into the circulation, on the other hand, has been observed in many epithelial cell types and could therefore take place in the thyroid follicular cell [17]. There is some evidence from a proteomic analysis of exosomes in fetal bovine serum (FBS) that this occurs [4]. In this study, proteomic analysis of the exosome-enriched (EEM) and the exosome-free (EFM) fractions of bovine serum revealed that Tg was one of a total of 51 proteins found only in the EEM [4]. These results provide evidence that exosome-enclosed Tg is secreted through the basolateral aspect of the thyroid cell and into the general circulation.

In summary, we have demonstrated that Tg is secreted from FRTL-5 cells in culture in a membrane-enclosed form. Future studies aimed at identifying a mechanism by which Tg is diverted into an exosomal pathway, rather than transcytotic or lysosomal pathway, will be required to determine whether the Tg exosomal secretion we observed in monolayer cultures of rat thyroid cells occurs as a normal process* in vivo* and, if so, what function(s) Tg could perform within and outside of the thyroid gland.

## Figures and Tables

**Table 1:** Uncalibrated optical density (OD) measurements of the Tg bands from Figure 3 corresponding to exosome pellets are presented here. The “sample applied” refers to the total volume applied to the gel and “sample amount” refers to the volume of the original dissolved exosome pellet present within that applied sample. Optical density measurements are normalized to the sample volumes and the ratio of Tg optical density to total protein concentration of the exosome samples is calculated and represented here multiplied by a factor of 1000.

Treatment	OD	Sample applied (*μ*L)	Sample amount (*μ*L)	OD/sample	Protein (*μ*g/mL)	Ratio
5H culture 1	0.184	15	7.5	0.368	50.22	7.328
6H culture 1	0.261	15	7.5	0.522	36.69	14.227
5H culture 2	0.085	15	3.5	0.364	48.50	7.505
6H culture 2	0.134	15	3.5	0.574	36.82	15.589
